# Activation of GHSRs Facilitates the Excitability of Dentate Gyrus Granule Cells and Short‐Term Spatial Memory via Multiple Ionic and Signaling Mechanisms

**DOI:** 10.1002/adbi.70145

**Published:** 2026-08-04

**Authors:** Chidiebele S. Oraegbuna, Phani K. Kola, Simon L. Griggs, Saobo Lei

**Affiliations:** ^1^ Department of Biomedical Sciences School of Medicine and Health Sciences University of North Dakota Grand Forks North Dakota USA

**Keywords:** glutamate, memory, receptor, signal, synapse, transmitter

## Abstract

Ghrelin is the endogenous ligand of the ghrelin secretagogue receptors (GHSRs). Although the GHSRs are densely expressed in the dentate gyrus (DG) granule cells (GCs), their roles in the DG GCs remain to be fully understood. We probed the actions of ghrelin on the DG GCs with whole‐cell recordings and its effects on short‐term spatial memory using the Y‐maze spontaneous alternation test. Application of ghrelin excited the DG GCs by depressing the G protein‐coupled inwardly rectifying K^+^ (GIRK) channels and ATP‐sensitive K^+^ (K_ATP_) channels, activating the hyperpolarization‐activated cyclic nucleotide‐gated (HCN) channels and a TTX‐resistant, persistent Na^+^ channel. Adenylyl cyclase and EPAC2 were required, whereas phospholipase C and protein kinase A were not needed for the ghrelin‐mediated excitation of DG GCs. Overnight fasting augmented the excitability of the DG GCs via activation of GHSRs, suggesting a role for endogenously released ghrelin. Ghrelin also enhanced synaptic recruitment of action potentials in DG GCs. Microinjection of ghrelin into the DG increased the alternating sequences in the Y maze test via GHSR‐mediated elevation of cAMP and EPAC signals. Our results provide an action mode to explain the roles of GHSRs in improving cognitive functions.

## Graphical abstract

Ghrelin excites the dentate gyrus (DG) granule cells via GHSR‐mediated modulation of diverse ionic channels and activation of D_1_R, cAMP, and EPAC2. Similarly, overnight calorie restriction alters neuronal excitability in a ghrelin‐dependent manner. Infusion of ghrelin directly into the DG improves short‐term spatial memory in the Y‐maze, providing possible mechanisms for the role of GHSRs in learning and memory.

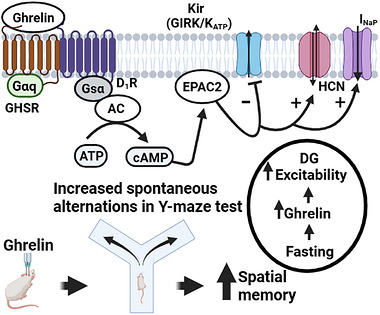

## 1 Introduction

Ghrelin is a 28‐amino‐acid peptide that is secreted mainly in the stomach, specifically by the X/A‐like cells [1]. For the maturation of the active form of ghrelin, the 117‐amino acid precursor undergoes proteolytic processing within the endoplasmic reticulum to form the 28‐amino‐acid peptide known as des‐acyl ghrelin [2]. Des‐acyl ghrelin is further acylated at Ser3 by the ghrelin O‐acyltransferase enzyme to generate the active form, acyl ghrelin [3, 4], which we denote as ghrelin thereafter. Ghrelin is expressed in the peripheral tissues, including the gastric mucosa, heart and kidney, as well as the CNS such as the pituitary and hypothalamus [1, 5, 6, 7]. The peripherally produced ghrelin can cross the blood–brain barrier [8, 9, 10, 11] to exert a variety of central effects. Ghrelin has been shown to regulate a myriad of physiological functions including growth hormone secretion [12], feeding‐associated behaviors [13, 14, 15], glucose homeostasis [16], energy balance [17], cardiovascular function [18], inflammation [19], mood [20], cognition and Alzheimer's disease [21, 22], and neuroprotection [23], although the underscoring cellular and molecular mechanisms of ghrelin have not been fully elucidated.

Ghrelin is the endogenous ligand for the ghrelin hormone secretagogue receptors (GHSRs), which have two isoforms: GHSR‐1α and GHSR‐1β [24]. GHSR‐1α is the active isoform of the receptors, while GHSR‐1β is the inactive analog. GHSRs (denoting GHSR‐1α thereafter) canonically signal via the Gq proteins, resulting in the activation of PLCβ [23]. However, the GHSRs can also induce a G‐protein coupling switch from Gq to Gs, leading to the activation of adenylyl cyclase to increase cyclic AMP levels [25, 26]. This switching is mainly because of the dimerization of GHSR‐1α with the D_1_ receptors [27, 28, 29]. The GHSRs are expressed in several brain regions including the hypothalamus, substantia nigra, ventral tegmental area and hippocampus [30, 31]. The dentate gyrus (DG) granule cells (GCs) of the hippocampus express a high density of GHSRs [30, 31], whereas the roles and underlying mechanisms of ghrelin in DG GCs have not been determined. In the current work, we probed the effects of ghrelin on the excitability of DG GCs and studied the roles of GHSRs in the DG in the modulation of spatial memory. Our results showed that activation of GHSRs excited DG GCs via depression of the G protein‐coupled inwardly rectifying K^+^ (GIRK) and ATP‐sensitive K^+^ (K_ATP_) channels and activation of the hyperpolarization‐activated cyclic nucleotide‐gated (HCN) channels and a persistent Na^+^ channel. Microinjection of ghrelin into the DG increased the alternating sequences in the Y maze test, indicating that GHSR‐mediated excitation of DG GCs augmented short‐term spatial memory. Elevation of intracellular cAMP levels and the activity of Exchange Protein directly Activated by cAMP (EPAC) were involved in ghrelin‐mediated excitation of DG GC and augmentation of spatial learning.

## 2 Materials and Methods

### 2.1 Ethical Statement

The procedures and experiments in this study were reviewed and approved by the Institutional Animal Use and Care Committee (IACUC) of the University of North Dakota and were conducted according to the National Institutes of Health's Guide for the Care and Use of Laboratory Animals.

### 2.2 Preparation of Hippocampal Slices

Hippocampal brain slices (330 µm) were prepared from C57BL/6J mice aged 18‐ to 28‐day‐old as previously described [32, 33]. Equal numbers of male and female animals were maintained across experiments as closely as possible. Mice were housed in the Center for Biomedical Research at the University of North Dakota with food and water available ad libitum. The animal rooms were maintained on a 14/10 h light–dark cycle (lights on at 7:00 a.m.), with a room temperature of 22°C. Mice were deeply anesthetized with isoflurane and decapitated for brain dissection. Brains were quickly removed and sectioned using a vibrating blade microtome (VT1200S; Leica) in an ice‐cold saline cutting solution containing (in mM) 250 sucrose, 120 N‐methyl‐D‐glucamine (NMDG), 19.2 NaHCO_3_, 2.8 KCl, 1 NaH_2_PO_4_, 2 CaCl_2_, 2.4 MgCl_2_ and 8 glucose, pH 7.3 (continuously saturated with 95% O_2_ and 5% CO_2_) [34]. The slices were subsequently incubated at 35°C for 30 min in the extracellular solution containing (mM): 60 sucrose, 124 NaCl, 23 NaHCO_3_, 3.3 KCl, 1.2 NaH_2_PO_4_, 2.4 CaCl_2_, 1.7 MgCl_2_ and 9.5 glucose (saturated with 95% O_2_ and 5% CO_2_). Following incubation, slices were kept at room temperature until use. All animal procedures conformed to the guidelines approved by the University of North Dakota Animal Care and Use Committee.

### 2.3 Electrophysiology

Whole‐cell patch‐clamp recordings were conducted in the DG GCs by utilizing a Multiclamp 700B amplifier (Molecular Devices) in voltage‐clamp or current‐clamp mode. Cells were visually identified with infrared video microscopy (BX51WI microscope, Olympus) and differential interference contrast optics [33, 34]. The temperature of the bath solution was maintained between 32°C and 34°C by an inline heater connected to an automatic temperature controller (TC‐324C, Warner Instruments). Recordings were performed in an extracellular solution containing (in mM): 130 NaCl, 24 NaHCO_3_, 3.5 KCl, 1.25 NaH_2_PO_4_, 2.5 CaCl_2_, 1.5 MgCl_2_, and 10 glucose, saturated with 95% O_2_ and 5% CO_2_. The intracellular recording pipettes were filled with a solution containing (in mM): 120 K^+^‐gluconate, 10 KCl, 5 NaCl, 2 MgCl_2_, 10 HEPES, 0.5 EGTA, 2 ATPNa_2_, 0.4 GTPNa, and 5 phosphocreatine (pH 7.3). Resting membrane potentials (RMPs) and holding currents held at −60 mV were recorded in the extracellular solution containing tetrodotoxin (TTX, 0.5 µM) to inhibit action potential (AP) firing. Kynurenic acid (1 mM) and picrotoxin (100 µM) were supplemented in the extracellular solution to block glutamate and GABA_A_ receptors, respectively. APs were elicited by injecting a series of positive currents ranging from 20 to 280 pA at an increment of 20 pA, and the resulting AP numbers were recorded in the presence of picrotoxin (100 µM) and kynurenic acid (1 mM). For the experiments assessing the synaptic recruitment of action potentials in DG GCs, EPSPs were recorded from DG GCs in current‐clamp mode by placing a stimulation electrode in the medial molecular layer to stimulate the perforant path in the extracellular solution containing picrotoxin (100 µM) to block GABAergic transmission. The internal solution was the above K^+^‐gluconate‐containing intracellular solution. The stimulation intensity was set to the level that produced ∼50% of the maximal EPSP amplitude. The stimulation frequency was set at 0.1 Hz. The number of APs was counted within 150 ms after the stimulation artifact. Ghrelin (100 nM) was diluted in the extracellular solution and bath applied. To minimize potential receptor desensitization from continuous agonist application, ghrelin was bath applied once per slice. Pharmacological inhibitors were applied to the cells either extracellularly through the bath solution or intracellularly via the recording pipettes. For extracellular application, slices were preincubated for at least 1 h to ensure complete diffusion of reagents into the cells and were continuously perfused with the same concentration of the inhibitors, unless stated otherwise. For intracellular application, we waited for at least 15 min following the formation of a whole‐cell configuration to ensure adequate diffusion of inhibitors into the cells. Data were filtered at 2 kHz, digitized at 10 kHz, acquired, and subsequently analyzed using pCLAMP 10.7 software (Molecular Devices).

### 2.4 Overnight Fasting

For the fasting experiments, littermate cohort mice of both sexes were divided into control and fasting groups (*n* = 2 mice/sex/group) to reduce basal variability. The fasting group was transferred to fresh cages before the onset of the dark period to ensure that no food remained in the cages (17:00–9:00), whereas control animals were placed in fresh cages with ad libitum access to food. Both groups had free access to water. Brains from fasted and fed animals were dissected out and sectioned using the previously stated technique. RMPs and APs elicited by the current‐injection protocol were recorded from the DG GCs in slices cut from the fasting and normally fed mice.

### 2.5 Stereotaxic Surgery, Microinjection and Histology

Procedures for surgery and cannula placement were referred to our previous publications [34, 35, 36] with modifications. Male and female mice (8–11 weeks) were deeply anaesthetized with 5% vaporized isoflurane, and anesthesia was maintained by 3% isoflurane using the Kent Scientific SomnoSuite low‐flow anesthesia system, while mice were placed in a stereotaxic frame (Stoelting Co., Wood Dale, IL, USA). Mice were bilaterally implanted with a guide cannula (26G, P1 Technologies Inc., Roanoke, VA, USA) targeting the dorsal DG with coordinates (AP: − 3.3 mm, ML: ± 2.5 mm, DV: – 2.3 mm) with modification from our previous publication [36]. Cannulae were secured with dental acrylic bonded to two stainless steel screws (2.4 mm, P1 Technologies Inc.) inserted into the skull. Dummy cannulae were screwed into the guide cannulae until microinjection to prevent occlusion. After the surgery, animals were allowed to recover for a period of 7–10 days during which they were habituated by daily handling. Saline, ghrelin, GHRP‐6, SQ22536, and HJC0350 (0.5 µL volume per side) were bilaterally injected into the DG through an internal cannula (33 G, P1 Technologies Inc.). Two Hamilton syringes connected to an automated pump (Harvard Apparatus, Holliston, MA, USA) via polyethylene tubing were used to administer drug injections at a rate of 0.1 µL/min. After drug injection, the internal cannulae were left in place for ∼2 min to ensure ample diffusion. Following experiments, animals were anesthetized and bilaterally injected with 1% Chicago Sky Blue 6B (Sigma–Aldrich, St. Louis, MO, USA) into the DG to confirm cannula location. Mice were intracardially perfused with 0.01 M phosphate buffer saline (PBS) followed by 4% paraformaldehyde (4% PFA in 0.01 M PBS). The brains were taken out and kept in 4% PFA for 24 hrs. Coronal brain sections of 80 µm thickness were cut on a vibratome (VT1200; Leica Biosystems Inc.). Slices were stained with 0.1% cresyl violet prepared in acetic acid for 10–15 min to verify cannula tract. Animals with incorrect cannula placement were excluded from analysis.

### 2.6 Y Maze Spontaneous Alternation Test

The Y‐maze apparatus is made of gray‐colored plastic with three arms. The floor was 10 cm in width, each arm was 35 cm long, and the walls were 20 cm in height. Arms were randomly designated as either A, B, or C at an angle of 120° between each of the two arms. Prior to initiating the behavioral tests, animals were allowed to acclimate in the testing room for a minimum of 30 min. All animals were placed in the center of the apparatus and were allowed to explore all the arms for 5 min. The behavioral experiments were conducted between 12:00 and 16:00. The arms of the maze were thoroughly cleaned with 70% ethanol to remove residual odors between the animals. Animal movements were tracked using the ANY‐maze video tracking software (Stoelting Co.). The total number of arm entries and spontaneous alternations made were recorded, i.e., making consecutive entries in the following order: ABC, ACB, BCA, BAC, CAB, CBA. The percentage of spontaneous alternations made was calculated as follows:

$$\begin{array}{c} \%\;\mathrm{Alternations}=\mathrm{Total}\;\mathrm{number}\;\mathrm{of}\;\mathrm{alternations}\\ /\;\left(\mathrm{Total}\;\mathrm{arm}\;\mathrm{entries}-2\right)\;\times 100 \end{array}$$

### 2.7 Data Analysis

Data are presented as the mean ± SEM. “N” numbers in the text refer to the number of cells used for each *ex vivo* experiment and the number of animals used for the in vivo study. Wilcoxon matched‐pairs signed‐rank test (abbreviated as “Wilcoxon test” in the text), Mann–Whitney test, one‐way ANOVA followed by Dunnett's or Tukey's multiple‐comparisons test, or two‐way repeated‐measures ANOVA followed by a Sidak multiple‐comparisons test was used as appropriate for statistical analysis. For normally distributed data, dual comparisons were made by Student's *t*‐test. Net differences in spontaneous alternations were assessed using a one‐way ANOVA followed by Tukey's post‐hoc test. *p*‐values were reported throughout the text and significance was set at *p* < 0.05. For all experiments, data from male and female mice were analyzed separately and pooled together if there were no significant differences. Statistical analysis was performed using Graphpad Prism or Origin 7.

## 3 Results

### 3.1 Activation of GHSRs Excites DG GCs

**FIGURE 1:**
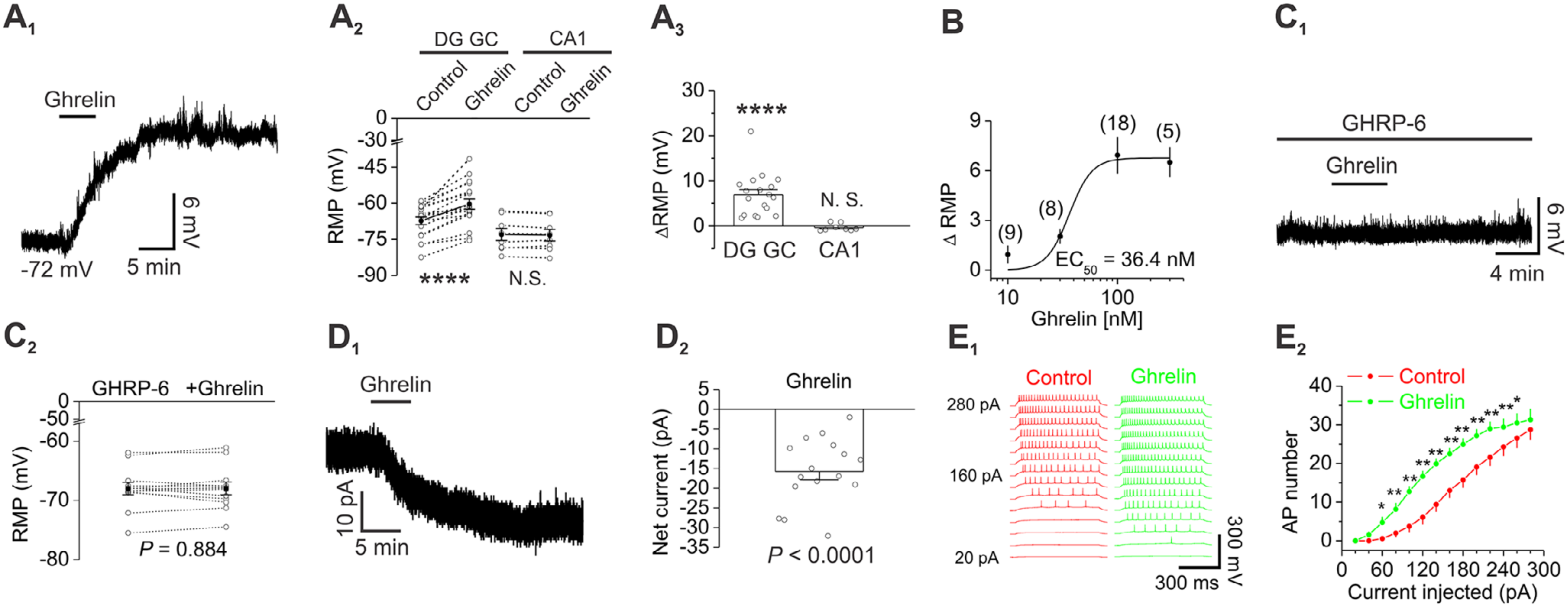
Activation of GHSRs excites DG GCs. (A_1_‐A_3_) Bath application of ghrelin (100 nM) induced subthreshold depolarization of DG GCs. (A_1_) RMP recorded from a DG GC in response to bath application of ghrelin. (A_2_) *Left*: Summary graph showing the RMPs recorded from the same population of DG GCs prior to and after application of ghrelin. *Right*: Summary graph showing RMPs recorded from the same population of CA1 pyramidal neurons prior to and after application of ghrelin. The empty circles and dashed lines represent data from individual cells, and the filled circles and the solid line are their averages. (A_3_) Summary graph showing ghrelin‐elicited net depolarization in DG GCs without effect on CA1 pyramidal neurons. (B) Concentration‐response curve of ghrelin. The numbers inside the parentheses are the numbers of cells recorded at each concentration. (C_1_‐C_2_) Pretreatment of slices with the selective GHSR antagonist, GHRP‐6 (1 µM), blocked ghrelin‐elicited depolarization. (C_1_) RMP recorded from a DG GC in a slice treated with GHRP‐6 in response to bath application of ghrelin. (C_2_) Summary graph showing that pretreatment of slices with and continuous bath application of GHRP‐6 blocked ghrelin‐elicited depolarization of DG GCs. (D_1_‐D_2_) Bath application of ghrelin induced an inward current recorded at −60 mV. (D_1_) Current trace recorded from a DG GC prior to, during, and after the application of ghrelin. (D_2_) Summary graph denoting the inward currents induced by ghrelin. (E_1_‐E_2_) Application of ghrelin enhanced AP firing numbers elicited by injections of a series of positive currents. (E_1_) APs evoked by injections of positive currents from 20 pA to 280 pA at an increment of 20 pA in a DG GC prior to and after application of ghrelin. (E_2_) Summary graph showing that ghrelin increased the AP numbers (F_(1,11)_ = 37.51, *n* = 12, *p* < 0.0001, Two‐way repeated measures ANOVA followed by Sidak's multiple comparison test). N. S., no significance * *p* < 0.05, ** *p* < 0.01, **** *p* < 0.0001.

Because high densities of GHSRs have been detected in the DG GCs [30, 31, 37], we probed the effects of GHSR activation on the excitability of DG GCs. The extracellular solution contained kynurenic acid (1 mM) to block glutamatergic transmission and bicuculline (10 µM) to block GABAergic transmission. The intracellular solution was the potassium‐gluconate intracellular solution. Under these circumstances, the effects of ghrelin should be derived from the activation of GHSRs in the DG GCs. Bath application of ghrelin (100 nM) induced a subthreshold depolarization of DG GCs (Control: −67.3 ± 1.6 mV, Ghrelin: −60.3 ± 2.1 mV, net depolarization: 6.9 ± 1.1 mV, *n* = 18, *p* < 0.0001, Wilcoxon test, Figure 1A_1_‐A_3_). Ghrelin‐elicited depolarization was not sex‐dependent, as analysis of the data by sexes of the mice did not show significant differences (Males: Control: −67.1 ± 2.5 mV, Ghrelin: −60.6 ± 2.9 mV, net depolarization: 6.5 ± 1.0, *n* = 10, *p* = 0.0001; Female: Control: −67.4 ± 1.9 mV, Ghrelin: −59.9 ± 3.3 mV, net depolarization: 7.5 ± 2.2 mV, *n* = 8, *p* = 0.012; Male vs. Female: F_(1,7)_ = 0.132, *p* = 0.727). We therefore used mice of both sexes, and the number of males and females was kept as equal as possible for the remaining experiments. The EC_50_ of ghrelin was calculated to be 36.4 nM (Figure 1B). We therefore used ghrelin at 100 nM for the remaining experiments because this is a near‐saturating concentration.

We further tested whether ghrelin‐induced depolarization was mediated by activation of GHSRs by performing the following two experiments. First, we tested whether ghrelin exerted any depolarization by recording from the pyramidal neurons of the CA1 region, where there is no expression of GHSRs in mice [30]. Bath application of the same concentration of ghrelin (100 nM) failed to induce significant depolarization (Control: −72.9 ± 2.5 mV, Ghrelin: −73.3 ± 2.4 mV, net depolarization: −0.3 ± 0.3 mV, *n* = 8, *p* = 0.308, Figure 1A_2_‐A_3_). Second, we tested the involvement of GHSRs by applying the selective antagonist, [D‐Lys^3^]‐GHRP‐6 (denoted as GHRP‐6 thereafter) [38, 39]. As it takes time for the peptide antagonists to permeate slice tissue to fully occupy and block GHSRs, we pretreated slices with and continuously bath‐applied GHRP‐6 (1 µM). Treatment of slices with GHRP‐6 alone did not significantly alter the basal levels of RMPs (Control: −67.3 ± 1.6 mV, *n* = 18, GHRP‐6: −68.0 ± 1.0 mV, *n* = 12, F_(1,11)_ = 0.416, *p* = 0.532, Figure 1A_2_, C_2_), suggesting that the level of endogenously released ghrelin is insufficient to exert significant depolarization of DG GCs in normally fed mice. However, treatment of slices with GHRP‐6 blocked ghrelin‐induced depolarization (GHRP‐6: −68.0 ± 1.0 mV, GHRP‐6 + ghrelin: −67.9 ± 1.1 mV, *n* = 12, *p* = 0.884, Figure 1C_1_‐C_2_), indicating that ghrelin depolarizes DG GCs by activation of GHSRs.

In voltage clamp at −60 mV, application of ghrelin elicited an inward current (−15.8 ± 2.1 pA, *n* = 16, *p* < 0.0001, Figure 1D_1_‐D_2_). Because the DG GCs did not show spontaneous firing at their resting membrane potentials, we probed the effects of GHSR activation on action potential firing by injecting a series of positive currents from 20 pA to 280 pA. Bath application of ghrelin significantly increased the firing frequency (F_(1,11)_ = 37.51, *n* = 12, *p *< 0.0001, Figure 1E_1_‐E_2_), indicating that activation of GHSRs excites DG GCs.

### 3.2 GHSR Activation Increases the Input Resistance and Augments Time Constants of DG GCs

**FIGURE 2:**
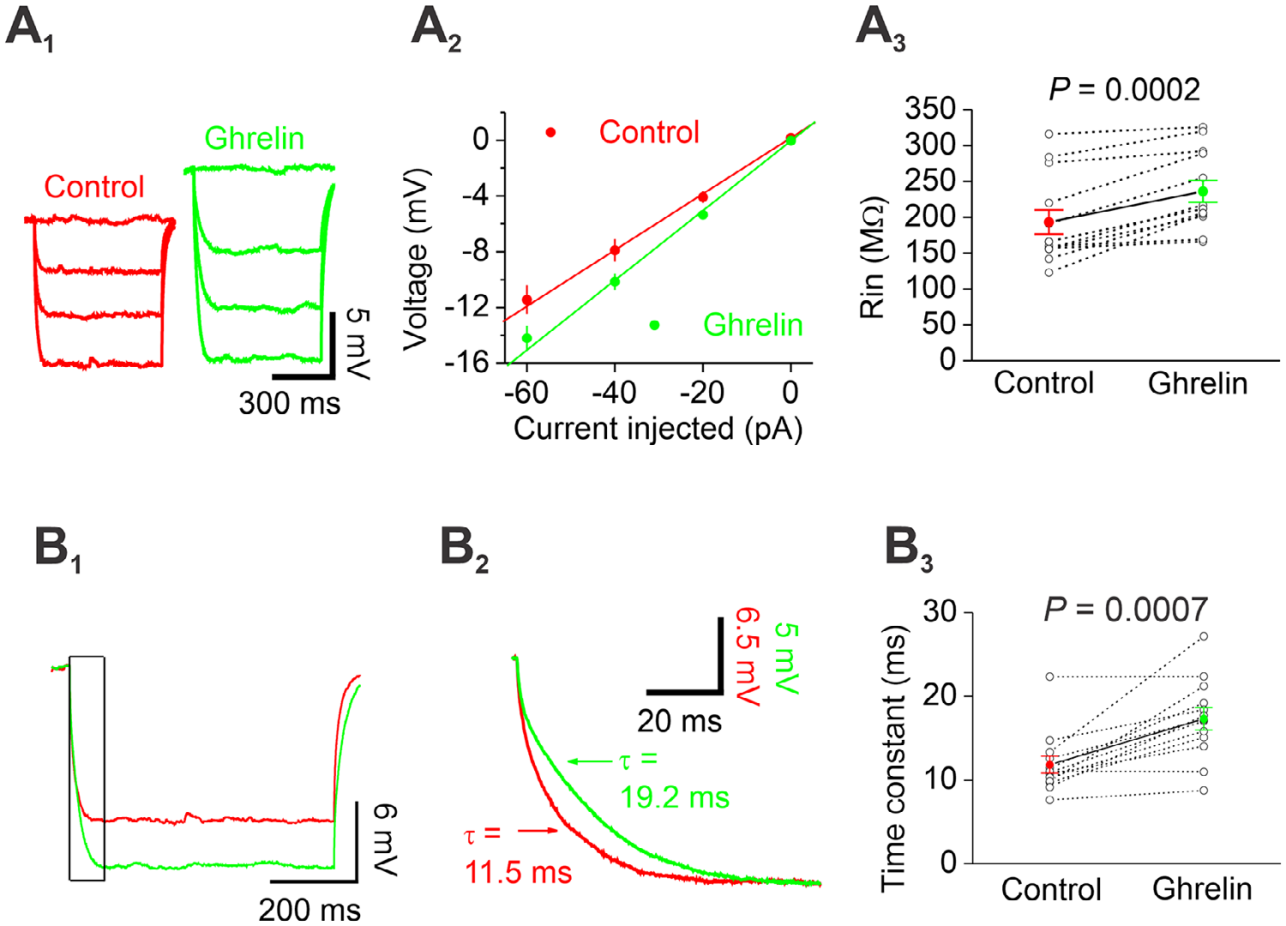
Ghrelin increases the input resistance and time constants of the DG GCs. (A_1_‐A_3_) Ghrelin enhanced the input resistance of GCs. (A_1_) Voltage responses evoked by injecting negative currents from 0 pA to −60 pA at an interval of 20 pA prior to (*red*) and after (*green*) application of ghrelin from a GC. (A_2_) Current–voltage relationship averaged from 13 cells recorded. Input resistance was obtained by performing a linear fit of the current–voltage relationship. (A_3_) Summary graph for input resistance prior to and after the application of ghrelin (*n* = 13). (B_1_‐B_3_) Ghrelin increased the time constants of GCs. (B_1_) Voltage response evoked by injection of −60 pA prior to (red) and after (*green*) application of ghrelin. (B_2_) Expansion of the voltage transient shown in the box in “B_1_” to indicate ghrelin‐induced augmentation of membrane time constants. (B_3_) Summary graph for membrane time constants prior to and after the application of ghrelin (*n* = 13).

We tested whether ghrelin alters the passive properties of DG GCs. Bath application of ghrelin increased the input resistance (Rin) obtained by injecting negative currents from 0 to −60 pA with 20 pA steps for a duration of 600 ms prior to and after the application of ghrelin when the maximal effect of ghrelin was achieved. We fit the I–V relationship with a linear function for each cell to obtain Rin, which equals the slope of the linear fit (Figure 2A_1_‐A_2_). Application of ghrelin significantly increased Rin (Control: 193 ± 17 MΩ, Ghrelin: 236 ± 15 MΩ, *n* = 13, *p* = 0.0002, Figure 2A_3_). Ghrelin also increased the membrane time constants obtained by fitting a single exponential function to the voltage transient elicited by −60 pA current step (Control: 11.8 ± 1.0 ms, Ghrelin: 17.3 ± 1.3 ms, *n* = 13, *p* = 0.0007, Figure 2B_1_‐B_3_).

### 3.3 GHSR Activation Excites DG GCs by Depressing Inwardly Rectifying K^+^ Channels and Activating Cation Channels

**FIGURE 3:**
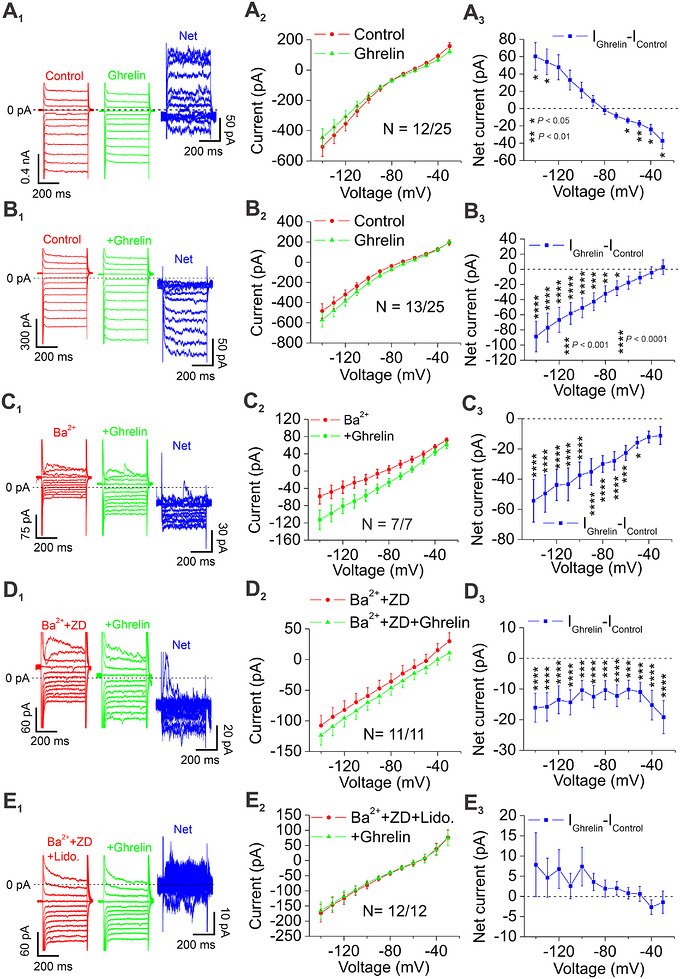
Ghrelin excites the DG GCs by depressing inwardly rectifying K^+^ channels and activating cation channels. (A_1_‐A_3_) Ghrelin‐induced excitation of DG GCs was mediated by depression of inwardly rectifying K^+^ channels. (A_1_) Currents elicited by a voltage step protocol before (*red*) and during (*green*) bath application of ghrelin and the net current obtained by subtraction (*blue*) from a GC. Cell was held at −60 mV and stepped from −140 to −30 mV for 400 ms at a voltage interval of 10 mV every 10 s. Steady‐state currents were measured within 5 ms prior to the end of the step voltage protocols. Note the difference in the scale bars. The dashed line was the zero current level (*n* = 12). (A_2_) I–V curve averaged from 12 GCs before and after the application of ghrelin. (A_3_) I–V curve of the net current obtained by subtracting the currents in the control condition from those after the application of ghrelin. Note that the net currents showed inward rectification, suggesting the involvement of Kir channels. (B_1_‐B_3_) Ghrelin‐elicited excitation is mediated by activating cation channels. (B_1_) Currents recorded from a GC in response to a voltage step protocol before (*red*) and after (*green*) bath application of ghrelin and the net current obtained by subtraction (*blue*) (*n* = 13). (B_2_) I–V curves of the currents elicited by the voltage‐step protocol before and after application of ghrelin. (B_3_) Net current obtained by subtracting currents in the control condition from those recorded in the presence of ghrelin. (C_1_‐C_3_) In the extracellular solution containing Ba^2+^ to block Kir channels, ghrelin‐elicited currents showed an I–V curve resembling cation channels in 7 out of 7 cells examined. (C_1_) Currents recorded from a GC in the presence of Ba^2+^ in response to a voltage step protocol before (*red*) and after (*green*) bath application of ghrelin and the net current obtained by subtraction (*blue*) (*n* = 7). (C_2_) I–V curves of the currents generated by the voltage‐step protocol before and after the application of ghrelin in the presence of Ba^2+^. (C_3_) Net currents obtained by subtracting currents in the presence of Ba^2+^ alone from the currents generated in the presence of Ba^2+^ and ghrelin. (D_1_‐D_3_) Ghrelin excited the DG GCs by activating the HCN channels. (D_1_) Currents recorded from a GC in response to a voltage step protocol before (*red*) and after (*green*) bath application of ghrelin and the net current obtained by subtraction (*blue*) in the presence of extracellular Ba^2+^ and intracellular ZD7288 (*n* = 11). (D_2_) I–V curves of the currents generated by the voltage‐step protocol before and after the application of ghrelin in the presence of Ba^2+^ and ZD7288. (D_3_) Net currents acquired by subtracting the currents in the presence of Ba^2+^ and ZD7288 from the currents in the presence of Ba^2+^, ZD7288, and ghrelin. (E_1_‐E_3_) Ghrelin activates a TTX‐resistant persistent sodium channel. (E_1_) Currents recorded from a GC in response to a voltage step protocol before (*red*) and after (*green*) bath application of ghrelin and the net current obtained by subtraction (*blue*). The extracellular solution ubiquitously contained Ba^2+^ and lidocaine, and the intracellular solution contained ZD7288. (E_2_) I–V curves of currents recorded from the same neuron before and after the application of ghrelin in the presence of Ba^2+^and ZD7288 and lidocaine. (E_3_) Net currents obtained by subtracting the currents in the presence of Ba^2+^, ZD7288, and lidocaine from those with Ba^2+^, ZD7288, lidocaine, and ghrelin. ****p < 0.0001, ***p < 0.001, **p < 0.01, *p < 0.05, two‐way repeated measures analysis of variance followed by Šídák's multiple comparisons test.

We further measured the I–V relationship of ghrelin‐elicited currents to probe the ionic mechanisms whereby activation of GHSRs excites DG GCs. Cells were held at −60 mV and stepped from −140 to −30 mV for 400 ms at a voltage interval of 10 mV every 10 s. Steady‐state currents were measured within 5 ms prior to the end of the step voltages. Out of 25 cells recorded, the I‐V curve of ghrelin‐elicited currents from 12 cells showed inward rectification with a reversal potential of −84.8 ± 4.3 mV (*n* = 12), close to the calculated K^+^ reversal potential, suggesting that activation of GHSRs depresses inwardly rectifying K^+^ (Kir) channels (Figure 3A_1_‐A_3_). The remaining 13 cells displayed an I–V curve resembling that of cation channels (*n* = 13; Figure 3B_1_‐B_3_). Because micromolar concentration of Ba^2+^ has been shown to block Kir channels [40, 41, 42], we included BaCl_2_ (500 µM) in the extracellular solution to block Kir channels [43] and constructed the I–V relationship in this condition. In the presence of TTX (0.5 µM) and Ba^2+^, the ghrelin‐elicited currents showed an I–V curve resembling the HCN channels in 7 out of 7 cells examined (Figure 3C_1_‐C_3_), suggesting that activation of HCN channels contributes to ghrelin‐elicited excitation of DG GCs. We further constructed the I–V curve in the extracellular solution supplemented with TTX (0.5 µM) and Ba^2+^ (500 µM) and the intracellular solution including the selective HCN channel blocker, ZD7288 (50 µM) [40]. Under these circumstances, the ghrelin‐induced currents showed an I–V curve resembling the TTX‐resistant, persistent Na^+^ channel (I_NaP_) in 11 out of 11 cells examined (Figure 3D_1_‐D_3_). This result suggests that activation of I_NaP_ is another mechanism that is similar to the ionic mechanism underlying neuronal excitation mediated by other G protein‐coupled receptors [44, 45, 46, 47]. Because lidocaine has been shown to block the TTX‐resistant I_NaP_ channels [48, 49, 50], we added lidocaine (1 mM), TTX (1 µM), and Ba^2+^ (500 µM) in the extracellular solution, and the intracellular solution contained ZD7288 (50 µM). Under these circumstances, application of ghrelin failed to induce significant currents in 12 out of 12 cells recorded (Figure 3E_1_‐E_3_).

### 3.4 Both GIRK and ATP‐Sensitive Potassium Channels Are Involved in GHSR‐Induced Excitation of DG GCs

**FIGURE 4:**
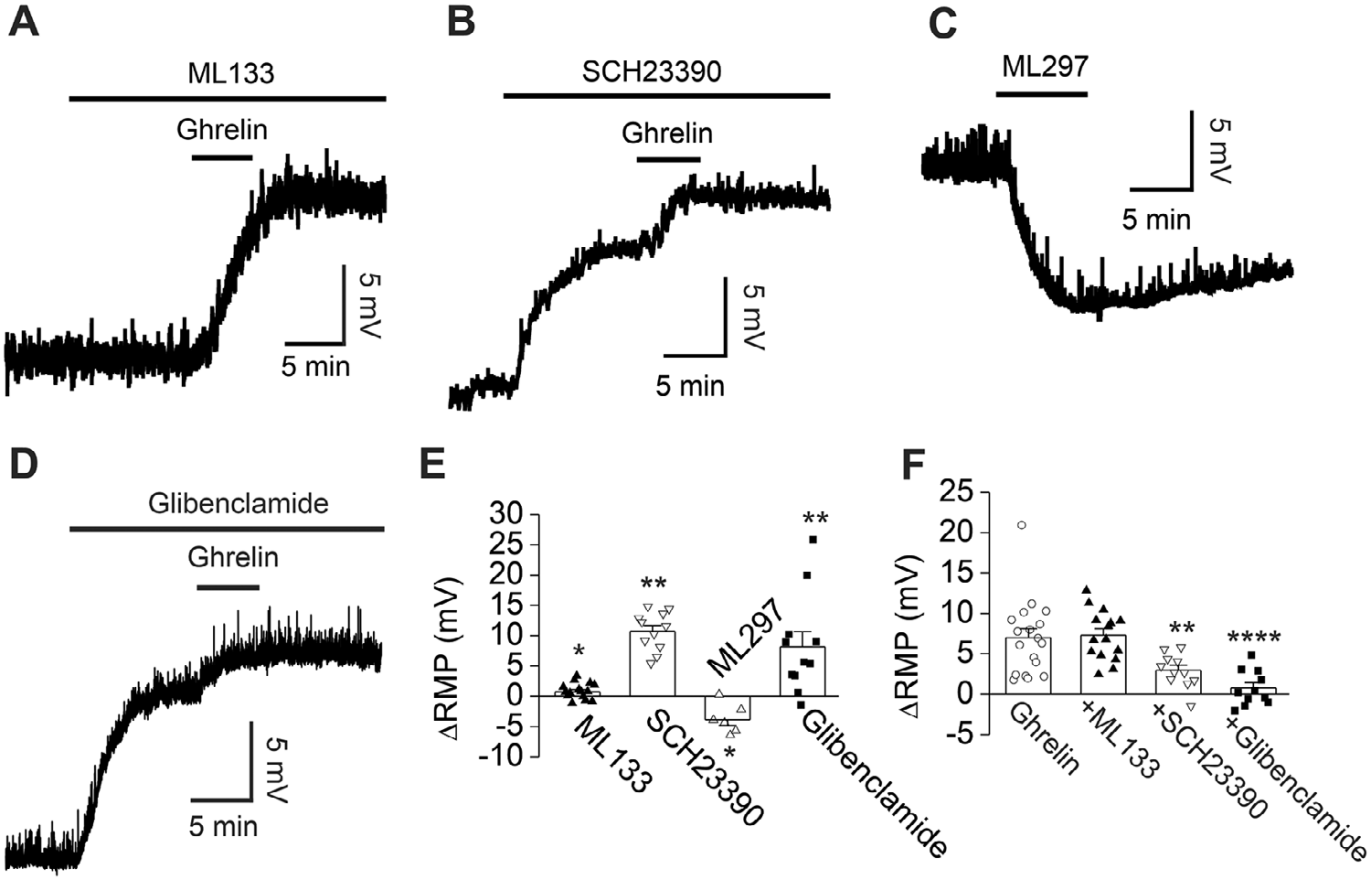
GIRK and K_ATP_ channels are involved in GHSR‐induced excitation of DG GCs. (A) RMP recorded from a DG GC in response to bath application of ML 133 (30 µM), a blocker of Kir2 subfamily channels, and co‐application of ML 133 and ghrelin. (B) Bath application of SCH23390 (20 µM) by itself induced a depolarization and decreased ghrelin‐induced subsequent depolarization. (C) Application of ML 297 (10 µM), the GIRK‐1 channel activator, resulted in the hyperpolarization of the cells. (D) Bath application of glibenclamide (10 µM), a K_ATP_ channel blocker, alone induced depolarization and reduced ghrelin‐mediated subsequent depolarization. (E) Summary data showing the effects of Kir channel modulators on RMPs of DG GCs. * *p* < 0.05, ** *p* <0.01 vs. baseline. (F) Summary data showing ghrelin‐induced depolarization in the presence of Kir channel blockers. ** *p* < 0.01, **** *p* < 0.0001 vs. ghrelin, one‐way ANOVA followed by Dunnett's test.

Kir channels comprise Kir2, Kir3 (GIRK) and Kir6 (ATP‐sensitive, K_ATP_) subfamilies and some K^+^ transport channels [51]. We further identified the type(s) of Kir channels involved in ghrelin‐mediated excitation of DG GCs. We included ZD7288 (50 µM) in the intracellular solution to block HCN channels and lidocaine (1 mM) in the extracellular solution to block I_NaP_ channels. Under these circumstances, application of ML133, a specific blocker for Kir2 subfamily [52, 53, 54, 55, 56], elicited little depolarization (0.85 ± 0.34 mV, *n* = 15, *p* = 0.041 vs. baseline, Figure 4A,E). In the presence of ML133, application of ghrelin induced a comparable depolarization (7.3 ± 0.8 mV, *n* = 15, *p* = 0.988 vs. ghrelin alone, Figure 4A,F), indicating that Kir2 subfamily is not the type of Kir channels involved in GHSR‐elicited excitation of DG GCs. We then tested the roles of GIRK channels in GHSR‐induced depolarization. Bath application of the selective GIRK channel blocker SCH23390 (20 µM) [42, 57, 58] by itself induced significant depolarization (10.8 ± 1.0 mV, *n* = 11, *p* = 0.001 vs. baseline, Figure 4B,E) and significantly reduced ghrelin‐elicited depolarization (2.9 ± 0.6 mV, *n* = 11, *p* = 0.01 vs. ghrelin alone, Figure 4B,F). In line with the participation of GIRK channels, application of ML297 (10 µM), an activator of GIRK1‐containing channels [59], induced a significant hyperpolarization (−3.7 ± 1.0 mV, *n* = 6, *p* = 0.01 vs. baseline, Figure 4C,E), suggesting that GIRK1 channels are functionally expressed in the DG GCs and might be involved in GHSR‐elicited excitation of DG GCs. Furthermore, bath application of the K_ATP_ channel blocker, glibenclamide (10 µM), induced significant depolarization (8.3 ± 2.5 mV, *n* = 11, *p* = 0.003 vs. baseline, Figure 4D,E) and significantly reduced ghrelin‐induced depolarization (0.72 ± 0.65 mV, *n* = 11, *p* < 0.0001 vs. ghrelin alone, Figure 4D,F), suggesting that K_ATP_ channels are also involved in ghrelin‐induced depolarization.

### 3.5 GHSR‐Mediated Excitation of DG GCs Is Not Dependent on PLCβ, but Mediated by Adenylyl Cyclase and cAMP

**FIGURE 5:**
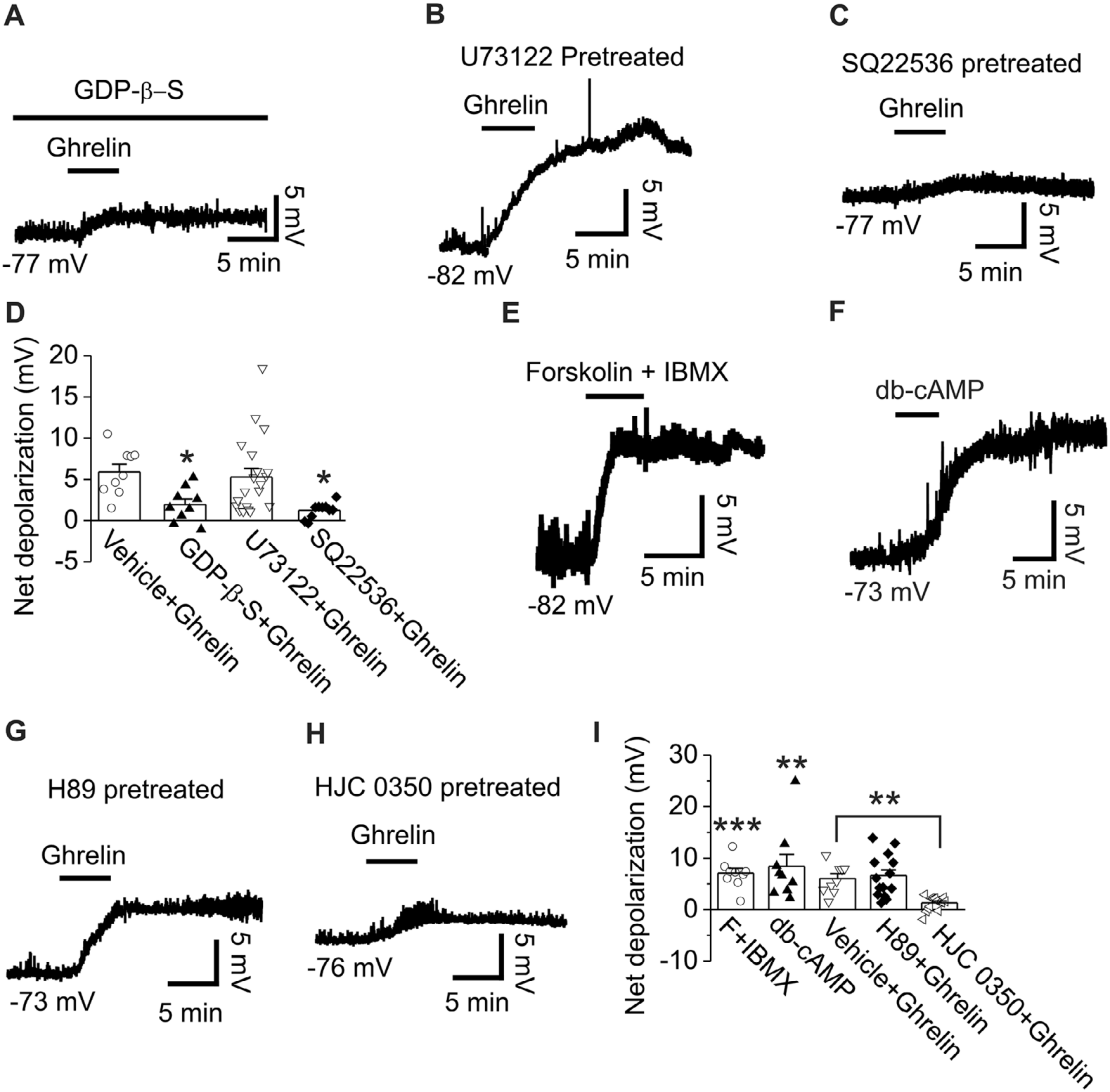
GHSR‐mediated excitation of DG GCs is not dependent on PLCβ, but mediated by adenylyl cyclase and cAMP. (A) Intracellular application of GDP‐β‐S (0.5 mM) via the recording pipettes reduced ghrelin‐mediated depolarization recorded from a DG GC. (B) Pretreatment of slices with the PLCβ inhibitor, U73122 (5 µM), failed to block ghrelin‐mediated depolarization. (C) Pretreatment of slices with SQ22536 (300 µM), an AC inhibitor, significantly reduced ghrelin‐elicited depolarization. (D) Summary graph showing the effects of G proteins, PLCβ and AC inhibitors on ghrelin‐mediated depolarization. * *p* < 0.05 vs. ghrelin, One‐way ANOVA followed by Dunnett's test. (E) Co‐application of the AC activator, forskolin (20 µM), and phosphodiesterase inhibitor, IBMX (200 µM), induced depolarization of a DG GC. (F) Bath application of the membrane‐permeant cAMP analog, db‐cAMP (0.5 mM), elicited depolarization of a DG GC. (G) Pretreatment of slices with the PKA inhibitor, H89 (10 µM), did not block ghrelin‐induced depolarization of a DG GC. (H) Pretreatment of slices with the EPAC inhibitor, HJC 0350 (50 µM), significantly reduced ghrelin‐induced depolarization of a DG GC. (I) Summary graph showing the roles of cAMP‐EPAC in ghrelin‐mediated excitation of DG GCs. * *p* < 0.05, ** *p* < 0.01, *** *p* < 0.001.

GHSRs are coupled canonically to the Gαq/11 proteins that signal through the PLCβ pathway [60]. We probed the role of the G proteins in ghrelin‐induced excitation of the DG GCs. Intracellular application of the G protein inactivator, GDP‐β‐S (0.5 mM) via the recording pipettes blocked GHSR‐mediated depolarization (Control: −75.2 ± 2.2 mV, Ghrelin: −73.8 ± 2.2 mV, net depolarization: 1.3 ± 0.8 mV, *n* = 10, *p* = 0.153, Figure 5A,D), suggesting that G proteins are involved in GHSR‐mediated excitation of DG GCs. We next tested the involvement of PLCβ in GHSR‐mediated excitation of DG GCs. Pretreatment of slices with and continuous bath application of the selective PLCβ inhibitor, U73122 (5 µM) failed to significantly alter ghrelin‐induced depolarization (Control: −67.7 ± 1.8 mV, Ghrelin: −62.4 ± 1.7 mV, net depolarization: 5.3 ± 1.0 mV, *n* = 20, *p* < 0.0001, Figure 5B,D), suggesting that GHSR‐mediated excitation of DG GCs is not mediated by PLCβ pathway. GHSRs are capable of coupling to Gαq, Gαi and Gαs [61, 62, 63]. As a plethora of evidence indicates that ghrelin signals through cAMP signaling [27, 28, 64, 65], we tested the roles of Gαs‐AC‐cAMP pathway in ghrelin‐mediated excitation of DG GCs. Pretreatment of slices with the selective AC inhibitor SQ22536 (300 µM) [40] abolished ghrelin‐mediated depolarization (Control: −64.2 ± 1.4 mV, ghrelin: −62.9 ± 1.3 mV, net depolarization: 1.2 ± 0.3 mV, *n* = 10, *p* = 0.525, Figure 5C,D), suggesting that AC is involved in ghrelin‐induced excitation of the GCs. In line with the role of AC, bath application of the AC activator, forskolin (F) (25 µM) together with the phosphodiesterase inhibitor, IBMX (200 µM), to elevate intracellular cAMP level elicited significant depolarization of DG GCs (Control: −69.7 ± 2.4 mV, F + IBMX: −62.7 ± 2.5 mV, net depolarization: 7.0 ± 2.8 mV, *n* = 9, *p* = 0.004, Figure 5E,I). Likewise, bath application of a membrane‐permeant cAMP analog, db‐cAMP (500 µM), elicited significant depolarization of DG GCs (Control: −65.0 ± 1.3 mV, db‐cAMP: −56.8 ± 2.0 mV, net depolarization: 8.3 ± 2.3 mV, *n* = 9, *p* = 0.008, Figure 5F,I). These results together indicate that GHSR‐mediated excitation of DG GCs is mediated by activation of Gαs‐AC‐cAMP pathway.

We further tested the roles of two cAMP targets, protein kinase A (PKA) and exchange protein directly activated by cAMP (EPAC), in ghrelin‐elicited excitation of DG GCs. Pretreatment of slices with and intracellular application of the PKA inhibitor H89 (10 µM) via the recording pipettes did not alter significantly ghrelin‐mediated depolarization (Control: −68.8 ± 1.6 mV, Ghrelin: −62.3 ± 1.9 mV, net depolarization: 6.5 ± 1.1 mV, *n* = 14, *p* < 0.0001, Figure 5G,I), suggesting that PKA is not required for GHSR‐mediated excitation of DG GCs. We next tested the roles of EPAC, as the DG expresses EPAC [66, 67]. Pretreatment of slices with the selective EPAC2 inhibitor, HJC0350 (50 µM) significantly reduced ghrelin‐induced depolarization (Control: −75.9 ± 2.5 mV, Ghrelin: −74.7 ± 2.4 mV, net depolarization: 1.2 ± 0.4 mV, *n* = 14, *p* = 0.006; *p* = 0.67 vs. Vehicle + Ghrelin, one‐way ANOVA followed by Dunnett's test, Figure 5H,I), indicating that EPAC2 is required for ghrelin‐induced excitation of DG GCs.

### 3.6 Overnight Fasting Excites DG GCs via Activation of GHSRs

**FIGURE 6:**
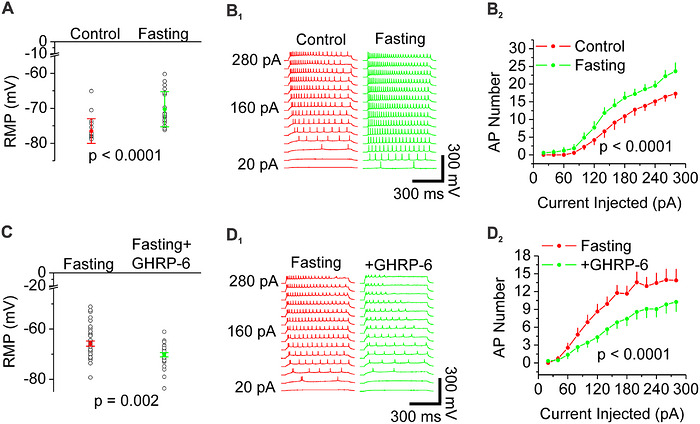
Overnight fasting induces depolarization and an increase in the action potential firing numbers of DG GCs. (A) Fasting induced depolarization of DG GCs. Resting membrane potential (RMP) recorded from the DG GCs in slices cut from normally fed mice (*n* = 6) and overnight fasting mice (*n* = 6). (B_1_‐B_2_) Fasting increases the action potential (AP) firing number of DG GCs. (B_1_) APs evoked by injections of a series of positive currents recorded from a DG GC in a slice cut from a normally fed mouse (red) and from a DG GC in a slice cut from a fasting mouse (green). (B_2_) Summary graph showing fasting‐induced increases in AP firing numbers (F_(1,364)_ = 45.04, *p* < 0.0001). (C) Incubation of the slices cut from fasted mice with the selective GHSR antagonist, GHRP‐6, induced hyperpolarization of DG GCs compared with the same populations of slices incubated in the normal extracellular solution. (D_1_‐D_2_) Treatment of the fasted slices with GHRP‐6 significantly decreased the AP firing numbers. Slices were cut from fasting mice and equally separated into two groups: fasting slices treated with normal extracellular solution and fasting slices treated with GHRP‐6. Recordings were alternately conducted from these two groups of slices. (D_1_) APs evoked by injections of the positive currents recorded from a DG GC in a fasting slice treated with normal extracellular solution (red) and from a DG GC in a fasting slice treated with GHRP‐6 in the extracellular solution (green). (D_2_) Summary graph showing that treatment of the fasting slices with GHRP‐6 significantly reduced the AP firing numbers (F_(1,658)_ = 50.79, *p* < 0.0001).

Circulating levels of ghrelin increase significantly during fasting and decrease following meal consumption [68, 69, 70]. We next tested the roles of endogenously released ghrelin in facilitating the excitability of DG GCs by fasting the mice overnight. The control and fasting groups contained equal male and female littermate cohort mice (*n* = 4/group). The fasting mice were deprived of food overnight (5:00 pm–9:00 am) with free access to water, whereas the fed group had ad libitum access to both food and water. Slices were prepared from the fasted and fed animals. We recorded RMPs and action potential numbers elicited by injection of positive currents from 20 pA to 280 pA at an interval of 20 pA from the DG GCs in slices cut from the 4 fasted and 4 fed mice (2 males and 2 females for each group) of the same cohorts. The RMPs recorded from the DG GCs in slices cut from fasted animals showed significant depolarization (Control: −76.5 ± 0.8 mV, *n* = 19, fasting: −70.3 ± 1.2 mV, *n* = 19, *p* < 0.001, Figure 6A). Furthermore, fasting also significantly increased the number of action potentials (F_(1,364)_ = 45.04, *p* < 0.0001, Figure 6B_1_‐B_2_). To confirm the role of ghrelin in fasting‐induced changes in excitability, we performed the experiment with its antagonist, GHRP‐6. We fasted 3 male and 3 female littermate cohort mice overnight. Slices prepared from each fasted mouse were equally divided into two groups, control slices incubated in normal extracellular solution and experimental slices incubated in the extracellular solution supplemented with the selective GHSR antagonist, GHRP‐6. We recorded the RMPs and AP firing numbers by injecting the abovementioned positive currents alternately into control slices and GHRP‐6‐treated slices. The rationale is that treatment of the fasted slices with the competitive GHSR antagonist would replace the binding of ghrelin to the GHSRs and annul the excitatory effects of ghrelin on DG GCs. Consistent with our anticipation, the RMPs in slices treated with GHRP‐6 (−70.3 ± 0.8 mV, *n* = 29) were significantly lower than those of the control slices (−66.9 ± 0.9 mV, *n* = 36, *p* = 0.002, Figure 6C). Likewise, the numbers of AP firing evoked by the positive currents in the fasted slices treated with GHRP‐6 were significantly smaller than those in the fasted slices kept in control extracellular solution (F_(1,658)_ = 50.79, *p* < 0.0001, Figure 6D_1_‐D_2_). Together, these results indicate that fasting‐induced increase in the excitability of DG GCs is mediated by endogenously released ghrelin.

### 3.7 Ghrelin Augments Synaptic Recruitment of Action Potentials in DG GCs

**FIGURE 7:**
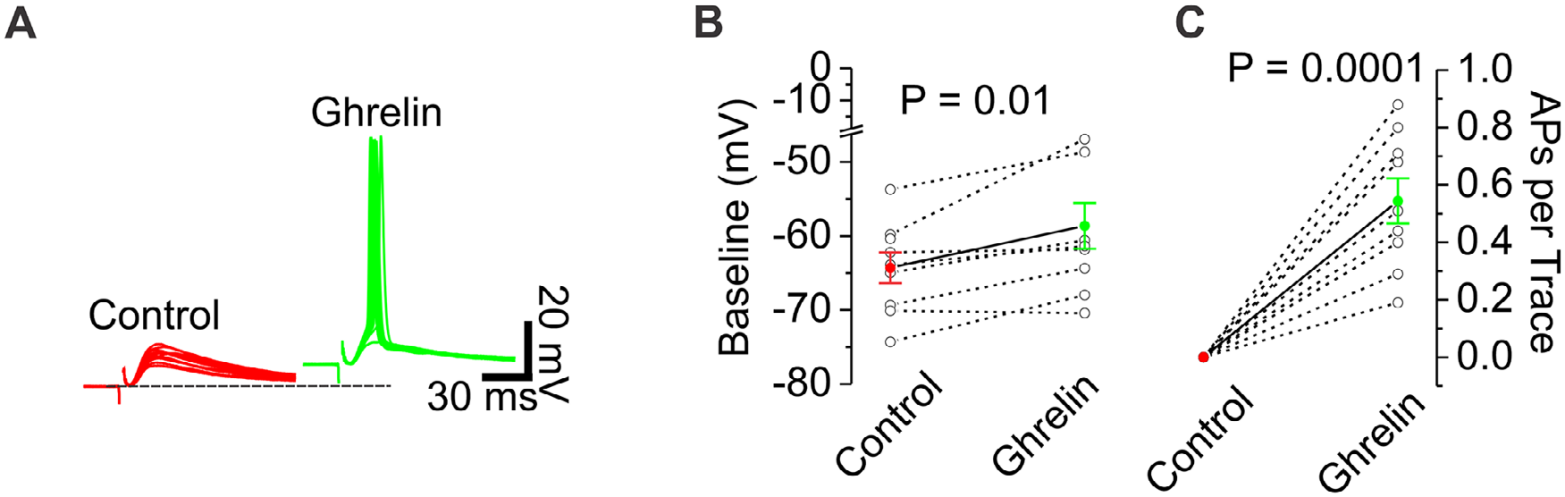
Ghrelin increased synaptic recruitment of action potentials in DG GCs at the perforant path‐GC synapses. (A) Left, Ten consecutive EPSPs recorded from a DG GC by placing a stimulation electrode in the medial molecular layer. Right, Ten EPSP/APs recorded from the same neuron after application of ghrelin. (B) Pooled data from 9 DG GCs showing that ghrelin induced depolarization of the baseline. (C) Summarized data showing that ghrelin enhanced the number of APs averaged from 10 consecutive traces per cell for 9 cells. Note that in the control condition, just EPSPs (not AP) were observed, and application of ghrelin initiated APs.

Our previous work indicates that activation of GHSRs facilitates glutamatergic transmission onto the DG GCs [28], and our current results suggest that GHSR activation increases the excitability of DG GCs. We next tested the effects of ghrelin on synaptic recruitment of action potentials in DG GCs by placing a stimulation electrode in the medial molecular layer to stimulate the perforant path at 0.1 Hz. EPSPs/APs were recorded from the DG GCs in current clamp with the abovementioned K^+^‐gluconate‐containing intracellular solution. Under these circumstances, bath application of ghrelin induced the depolarization of the baseline of the EPSPs (Control: −64.3 ± 2.1 mV, Ghrelin: −58.6 ± 3.1 mV, *n* = 9, *p* = 0.01; Figure 7A,B) and facilitated synaptic recruitment of action potentials in DG GCs (*n* = 9, *p* = 0.0001; Figure 7C). These results further confirm that activation of GHSRs facilitates glutamatergic excitation of DG GCs.

### 3.8 Ghrelin Facilitates Short‐Term Spatial Memory in the Y Maze Test

**FIGURE 8:**
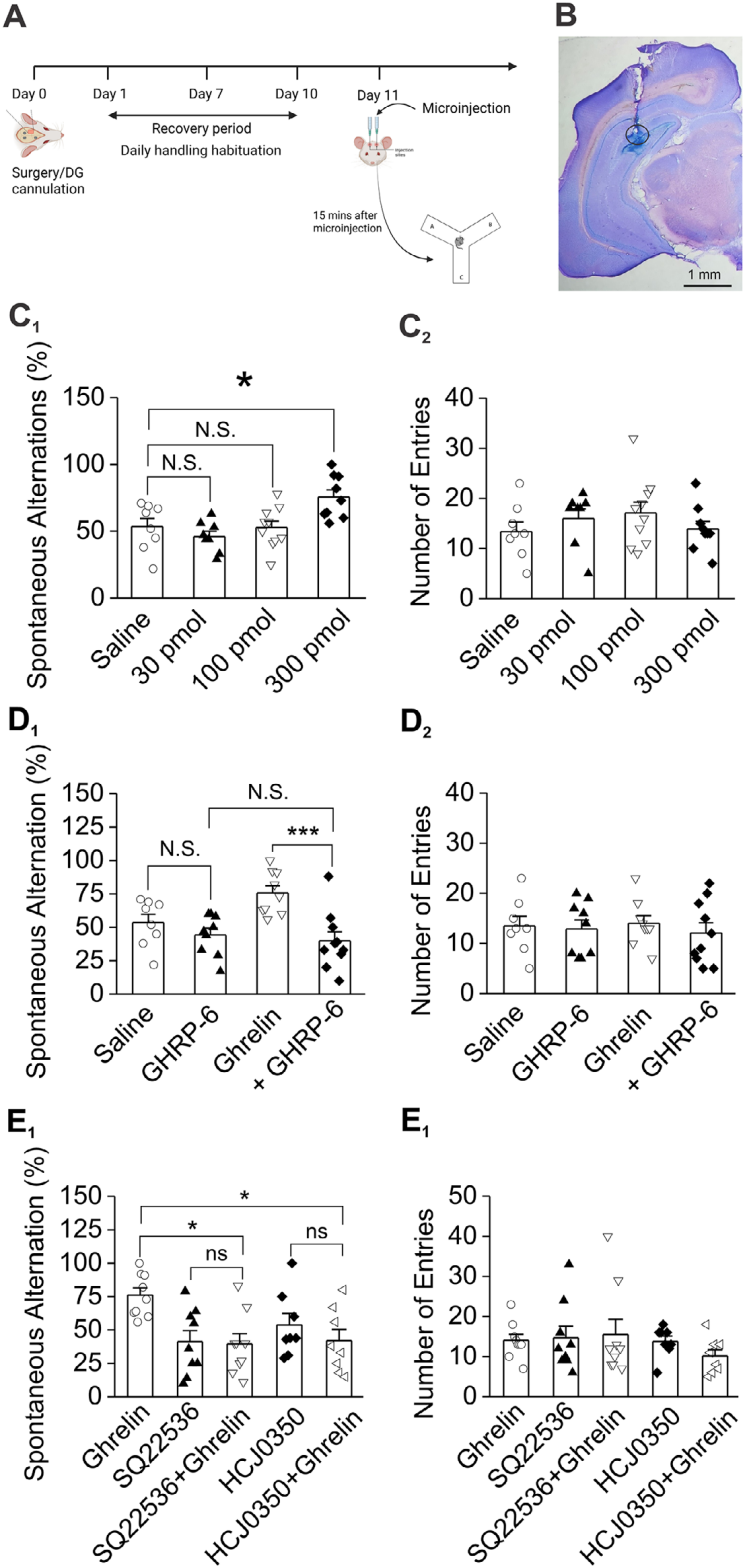
Microinjection of ghrelin into the DG facilitates short‐term spatial memory in the Y‐maze test by GHSR‐mediated AC‐cAMP‐EPAC signaling. (A) Schematic diagram showing the experimental procedures of the Y‐maze. Mice received daily handling after cannulation to habituate them to the experimental procedures for a minimum of 7 days. Drugs were microinjected into the DG of the mice 15 min prior to the behavioral experiments. (B) Microscopic photo to show the location of the cannula. Black circle highlights the cannula tip placement. (C_1_‐C_2_) Microinjection of ghrelin in the DG dose‐dependently increased spontaneous alternations without altering the number of arm entries. (C_1_) Direct microinjection ghrelin in the DG significantly potentiated spontaneous alternations at 300 pmol but had no effects at 30 pmol and 100 pmol. * *p* = 0.0265, one‐way ANOVA followed by Tukey's test. (C_2_) There was no significant difference in the number of arm entries in all groups. (D_1_‐D_2_) Prior microinjection of GHRP‐6 (5 nmol), the GHSR antagonist, abolished ghrelin‐elicited increases in spontaneous alternations. (D_1_) Microinjection of GHRP‐6 by itself did not alter spontaneous sequences, but blocked ghrelin‐induced increases in alternations. (D_2_) GHRP‐6 did not alter the number of arm entries. (E_1_‐E_2_) Prior microinjection of the AC inhibitor, SQ22536 (2 nmol) or EPAC inhibitor, HCJ0350 (1 nmol) blocked ghrelin‐induced enhancement of alternations (E_1_), but did not change the number of arm entries (E_2_).

Our previous results demonstrate that activation of GHSRs induced persistent elevation of glutamatergic transmission at the perforant path‐GC synapses [28], and the current results showed that ghrelin excited DG GC. We therefore probed the roles of ghrelin in short‐term spatial memory using the Y maze [71, 72]. We implanted cannulae bilaterally into the DG to probe the effects of GHSR activation on short‐term spatial memory [36]. Figure 8A is the timeline used for the experiments, and Figure 8B shows the location of a cannula tip for a representative experiment and the diffusion area of Chicago Sky Blue dye injected at the end of the experiments and the slice stained with cresyl violet acetate dye. Bilateral microinjection of ghrelin at the dose of 300 pmol into the DG significantly increased the spontaneous alternations in the Y maze compared with the mice injected with saline (*p* = 0.027, one‐way ANOVA followed by Tukey's test, Figure 6C_1_). However, microinjections at lower doses (30 nmol and 100 nmol) failed to significantly alter the spontaneous alternation sequences (30 nmol, *p* = 0.769; 100 nmol, *p* = 0.999; one‐way ANOVA followed by Tukey's test, Figure 8C_1_). Furthermore, ghrelin did not significantly alter the number of entries at the microinjected doses (F_(3,31)_ = 0.85, *p* = 0.477, Figure 8C_2_), suggesting that ghrelin does not alter the mobility of the mice. We microinjected ghrelin at 300 pmol for the remaining experiments to further study the mechanisms involved. Microinjection of the selective GHSR antagonist, GHRP‐6 (5 nmol) alone did not significantly alter the spontaneous alternation sequences compared with the mice injected with saline (*p* = 0.562, one‐way ANOVA followed by Tukey's test, Figure 8D_1_). However, subsequent microinjection of ghrelin in the presence of GHRP‐6 failed to increase spontaneous alternations in the Y‐maze (GHRP‐6 vs GHRP‐6 + Ghrelin; *p* = 0.861, one‐way ANOVA followed by Tukey's test, Figure 8D_1_), corroborating that ghrelin augments short‐term spatial memory via activation of GHSRs. There were no significant differences for the number of entries among these groups (F_(3,32)_ = 0.21, *p* = 0.889, Figure 8D_2_).

### 3.9 Adenylyl Cyclase and EPAC Are Involved in Ghrelin‐Mediated Augmentation of Short‐Term Spatial Memory

Because both AC and EPAC are involved in ghrelin‐mediated enhancement of glutamatergic transmission at the perforant path‐GC synapses [28] and excitation of DG GCs, we probed the roles of AC‐cAMP‐EPAC pathway in ghrelin‐mediated facilitation of short‐term spatial memory. Prior microinjection of the adenylyl cyclase inhibitor, SQ22536 (2 nmol), an effective dose that has been used previously [73], blocked ghrelin‐elicited enhancement of spontaneous alternation (*p* > 0.999, one‐way ANOVA followed by Tukey's test, Figure 8E_1_), suggesting that adenylyl cyclase is involved in ghrelin‐mediated augmentation of short‐term spatial memory. Likewise, prior microinjection of the EPAC inhibitor, HJC0350 (1 nmol), annulled the augmentation of spontaneous alternation elicited following microinjection of ghrelin (*p* = 0.832, one‐way ANOVA followed by Tukey's test, Figure 8E_1_) without altering the numbers of arm entries (F_(4,38)_ = 0.652, *p* = 0.629, one‐way ANOVA followed by Turkey's multiple comparison test, Figure 8E_2_), suggesting that EPAC is required for ghrelin‐evoked increases in short‐term spatial memory.

## 4 Discussion

Our results demonstrate that activation of GHSRs in the DG GCs induced increases in neuronal excitability. Ghrelin‐mediated increases in excitation of DG GCs are dependent on the activation of adenylyl cyclase and EPAC and mediated by depression of GIRK and K_ATP_ channels and concomitant activation of HCN and I_NaP_ channels. Activation of the GHSRs in the DG via microinjection of ghrelin improves short‐term spatial memory via cAMP and EPAC signals. Our results provide a cellular and molecular mechanism by which ghrelin facilitates neural network activities and cognition.

Whereas GHSRs are densely expressed in the DG GCs [30, 31, 37, 74, 75], the functions of GHSRs in the DG have not been determined. Our results showed that activation of GHSRs in the DG GCs augments the excitability of these neurons. Consistent with our results, previous work showed that peripheral injection of ghrelin increased the number of Fos‐positive neurons, as Fos is a marker of neuronal activity [76]. In the DG, ghrelin has been shown to increase adult neurogenesis [77, 78, 79, 80], which plays a role in cognition [81, 82]. As neural activities modulate neurogenesis [83], ghrelin‐mediated augmentation of neuronal activity in the DG GCs may play roles in neurogenesis and cognitive functions.

Increases in neuronal excitation can be mediated ionically by inhibiting K^+^ channels and/or by activating cation channels. Based on the voltage–current relationship of ghrelin‐sensitive currents, our data support the notion that both depression of Kir channels and activation of cation channels served as mechanisms responsible for GHSR‐mediated excitation of DG GCs. Kir channels are classified into four subfamilies: Kir2 (Kir2.1, Kir2.2, Kir2.3, and Kir2.4), Kir3 (GIRK channels, Kir3.1, Kir3.2, Kir3.3, and Kir3.4), Kir6 (K_ATP_ channels, Kir6.1, Kir6.2), and K^+^ transport channels (Kir1.1, Kir4.1, and Kir7.1) [51]. We further showed that GIRK and K_ATP_ channels are involved in GHSR‐elicited excitation of DG GCs. Consistent with our results, the DG expresses high densities of GIRK1‐3 with minimal levels of GIRK4 [84, 85], whereas K_ATP_ channels composed of Kir6.1/SUR1 or SUR2B subunits are detected in the DG GCs [33, 85, 86]. Functionally, ghrelin inhibited nociception‐activated GIRK channels in histaminergic neurons [87], and application of the selective K_ATP_ channel blocker, glibenclamide, blocked ghrelin‐induced depolarization in Neuropeptide Y neurons in the hypothalamus [88, 89, 90, 91], further supporting our conclusion that activation of GHSRs enhances neuronal excitability by depressing GIRK and K_ATP_ channels.

In addition to depressing GIRK and K_ATP_ channels, our findings showed that GHSR‐mediated excitation of DG GCs required the activation of two cation channels, including HCN and I_NaP_ channels. In line with our results, HCN channels are robustly expressed in the DG [92, 93, 94, 95]. The DG expresses HCN1, HCN2, and HCN4, with the expression of HCN3 being very negligible [93, 94, 96, 97]. TTX‐resistant persistent sodium channels, such as Nav 1.6 subtype, have been reported to be expressed in hippocampal granule cells and in the axons of the DG [98, 99]. Functionally, ghrelin abrogated methyl‐4‐phenylpyridinium‐mediated inhibition of HCN channel currents in dopaminergic neurons of the substantia nigra [100], suggesting that ghrelin facilitates HCN channels. In the arcuate nucleus and paraventricular nucleus, ghrelin increased the excitability of NPY/AgRP neurons by modulating several channels, including the HCN channels, to promote hunger signals [101]. Ghrelin has been shown to induce persistent inward currents in rat laterodorsal tegmental neurons that are insensitive to TTX [102]. GHSR interacts with receptors like the orexin receptors, which are known to elicit TTX‐insensitive inward currents [103, 104, 105, 106, 107]. All the evidence supports our results that activation of HCN and I_NaP_ channels is another ionic mechanism by which GHSR activation augments neuronal excitability.

In the current study, we included lidocaine in the extracellular solution to block I_NaP_ channels and isolate the effect of ghrelin on Kir channels (Figure 4), because few I_NaP_ channel blockers are available. QX314, a positively charged derivative of lidocaine, has been shown to block homomeric GIRK2 channels, without blocking GIRK channels that contain the GIRK1 subunit [108]. Application of the selective GIRK1 agonist, ML297, elicited significant hyperpolarization (Figure 4C,E), suggesting the type of GIRK channels involved in ghrelin‐mediated excitation of DG GCs contains GIRK1, which should be insensitive to lidocaine. Therefore, lidocaine‐mediated inhibition of GIRK channels in this study, if any, should be small. Indeed, application of SCH23390 in the extracellular solution containing lidocaine still induced significant depolarization (Figure 4B,E), suggesting there were still sizable GIRK currents remaining in the presence of lidocaine. One limitation of this study is that the ionic mechanisms are based on pharmacological data. Although our results indicate Ba^2^^+^, lidocaine, and SCH23390 altered ghrelin‐mediated currents significantly, suggesting the involvement of GIRK and I_NaP_, none of these inhibitors are specific. Future studies including molecular and genetic approaches will be used to further confirm the roles of the ion channels in ghrelin‐mediated excitation of DG GCs.

GHSRs canonically signal via the Gq proteins, resulting in the activation of PLCβ [23]. However, our results do not support a role of PLCβ in GHSR‐mediated excitation of DG GCs but indicate that cAMP and EPAC are necessary for ghrelin‐mediated excitation of DG GCs. We [28] and others [27, 104, 109, 110, 111, 112, 113, 114] have shown that GHSRs form heterodimers with the dopamine D1 receptor, which is a Gs‐coupled receptor. Our results support the action mode that heterodimerization of GHSR with the D_1_ receptor induces an active conformation in the GHSR that favors coupling to the Gαs pathway by occluding the Gαq protein. This mechanism has been used to explain the inhibitory effects on the Gi‐coupled cannabinoid receptor 1 when it dimerizes with the dopamine D_2_ receptor [115]. An alternative hypothesis is that GHSR interaction with the D_1_ receptor prevents rearrangement of the intracellular transmembrane loop necessary for recruiting the Gq protein to the receptor core, ultimately resulting in a shift to Gs protein signaling [116].

Our findings further show that ghrelin increased AP firing in response to EPSPs evoked in the DG GCs. Consistent with our results, ghrelin enhances synaptic recruitment of action potentials in DG GCs of rats [117]. It is important to note that ghrelin‐mediated effects on EPSP‐AP were recorded under inhibited GABAergic conditions. Therefore, our responses from ghrelin on AP firing should be interpreted within the context of low inhibitory input and may not be reflective of physiological conditions. Further studies using in vivo electrophysiology methods are therefore warranted.

As our results support the roles of GIRK, K_ATP_, HCN and I_NaP_ channels in ghrelin‐mediated excitation of DG GCs, it is reasonable to speculate that the cAMP/EPAC signals should modulate the functions of these channels. In line with our reasoning, cAMP and EPAC signals depress GIRK [118] and K_ATP_ [119, 120, 121, 122]. EPAC modulates HCN channels [123] and I_NaP_ [124]. EPAC specifically interacts with the sulfonylurea receptor 1 (SUR1), resulting in the closure of the K_ATP_ [125, 126]. Furthermore, EPAC‐mediated inhibition of SUR1 increases glutamate release and seizure susceptibility in dentate gyrus granule cells [127].

Neurogenesis contributes to the heterogeneity of GC maturation in the DG. Mature GCs have hyperpolarized RMPs and lower input resistance and excitability, whereas immature cells possess depolarized RMPs and higher input resistance and excitability [128, 129, 130, 131]. The discrepancy could be due to distinct expressions of G protein signals and the associated Kir channels, with the mature GCs expressing intact G protein signals and more Kir channels [128, 130]. The distinct developmental stages of DG GCs with the intrinsic Kir channels could explain the substantial variability of basal RMPs and excitabilities observed in DG GCs recorded. However, we consider that the variability of the basal level of RMPs in DG GCs is unlikely to affect ghrelin‐elicited excitation significantly, as we used the net depolarization evoked by ghrelin to limit the contribution of basal RMPs. The variability of the basal RMPs recorded from the DG GCs in slices cut from the mice undergone fasting is even larger (Figure 6C). Whereas treatment of GHRP‐6 significantly reversed the depolarized RMPs in fasting neurons (Figure 6C), suggesting that endogenously released ghrelin is an underlying mechanism, there could be another possibility, i.e., fasting altered the developmental stages of DG GCs, such as driving more immature cells into the DG, which displayed more depolarized RMPs and higher excitability. Consistent with this speculation, fasting promotes neurogenesis [132]. Nevertheless, the function of ghrelin could still be involved, because ghrelin promotes neurogenesis as well [79]. Thus, an alternative explanation for the effect of fasting is that fasting enhances the release of ghrelin, which further promotes neurogenesis, resulting in more immature cells with higher excitability in the DG. The heterogeneity of maturation of the DG GCs could also explain the disparity of the basal excitability of DG GCs in mice from different litters (Figure 6B_2_,D_2_).

Ghrelin‐deficient mice showed spatial learning impairments, and subsequent application of ghrelin rescued these deficits [133]. Disruption in the ghrelin gene results in cognitive impairments [134]. Under calorie restriction, ghrelin levels are elevated, leading to an improvement in cognitive performance [78, 135]. Although these data suggest a role for ghrelin in learning and memory, the exact cellular and molecular mechanisms whereby ghrelin facilitates memory have not been determined. Using the Y maze test, microinjection of ghrelin (30, 100 and 300 pMol) into the DG dose‐dependently increased the spontaneous alternating sequences significantly. Microinjection of the inhibitors for adenylyl cyclase and EPAC under separate conditions prior to microinjecting ghrelin led to a significant decrease in the alternating sequences. These results suggest that ghrelin‐mediated facilitation of short‐term spatial memory requires both adenylyl cyclase and Epac2. Our results therefore provide a basis to explain the roles of ghrelin in excitability and short‐term spatial memory.

## Author Contributions

**Chidiebele S. Oraegbuna**: experimental design and execution, analysis and manuscript drafting. **Phani K. Kola**: experimental execution, analysis. **Simon Griggs**: experimental execution. **Saobo Lei**: writing, revision, and editing .

## Conflicts of Interest

The authors declare no conflicts of interest.

## Data Availability

The data that support the findings of this study are available from the corresponding author upon reasonable request.
